# Use of the Micro-Agar Larval Development Test to Differentiate Resistant and Susceptible *Cooperia* spp. Isolates in Cattle Within the Context of Parasite Population Replacement

**DOI:** 10.3390/pathogens13110952

**Published:** 2024-10-31

**Authors:** Mariana Elisabet Fuentes, Mercedes Lloberas, Gisele Bernat, Eliana Riva, Milagros Junco, Silvina Fernández

**Affiliations:** 1 Departamento de Sanidad Animal y Medicina Preventiva, Facultad de Ciencias Veterinarias, Universidad Nacional del Centro de la Provincia de Buenos Aires, Tandil B7000, Buenos Aires, Argentina; eriva@vet.unicen.edu.ar; 2 Centro de Investigación Veterinaria de Tandil (CIVETAN), UNCPBA-CICPBA-CONICET, Tandil B7000, Buenos Aires, Argentina; gbernat@vet.unicen.edu.ar (G.B.); mjunco@vet.unicen.edu.ar (M.J.); 3 Instituto de Innovación para la Producción Agropecuaria y el Desarrollo Sostenible (IPADS Balcarce) EEA-INTA, Balcarce B7620, Buenos Aires, Argentina; lloberas.maria@inta.gob.ar; 4 Centro de Investigaciones en Sanidad Animal, Pública y Ambiental, Facultad de Ciencias Veterinarias, Universidad Nacional del Centro de la Provincia de Buenos Aires, Tandil B7000, Buenos Aires, Argentina

**Keywords:** anthelmintic resistance, micro-agar larval development test, gastrointestinal nematodes, *Cooperia*, cattle, in vitro tests, population replacement

## Abstract

Gastrointestinal nematode infections are a global concern in grazing cattle production systems, even more so due to the widespread problem of anthelmintic resistance. In response, early anthelmintic resistance detection methods, such as the micro-agar larval development test (MALDT), and parasite management strategies, such as the replacement of resistant parasite populations with susceptible ones, have been developed. This study aimed to characterize ivermectin-susceptible and -resistant isolates of *Cooperia* spp. using MALDT in the context of a parasite population replacement strategy. Three *Cooperia* spp. field isolates were evaluated: a susceptible one (*Coop-S*), a resistant one (*Coop-R*), and a post-replacement one (*Coop-PR*). The MALDT was performed in 96-well plates with 12 known concentrations of eprinomectin (EPR) on an agar base. Each test was performed in quadruplicate. Data analysis included nonlinear regression to determine EC50, EC90, and EC99 values, resistance ratios (RRs), and R^2^. The results showed clear differentiation between the isolates, with RR values of 5.78 and 1.28 for *Coop-R* and *Coop-PR*, respectively, compared to *Coop-S*. The MALDT proved to be a reliable tool for differentiating ivermectin-susceptible from ivermectin-resistant isolates of *Cooperia* spp., and future evaluations of this test in mixed nematode populations are recommended for routine diagnosis of anthelmintic resistance.

## 1. Introduction

Gastrointestinal nematode (GIN) infections are a global issue and significantly affect productive and reproductive parameters in bovine production systems, particularly in extensive pasture-based systems [1,2,3]. Before the development of anthelmintics, GIN infections were a clinical condition leading to high mortality rates, primarily in young stock [3,4]. However, with the advent of highly efficacious anthelmintic drugs, such as benzimidazoles and macrocyclic lactones, mortality rates have dropped and GIN infections have become mainly a subclinical disease. Subclinical GIN infections cause serious impacts on production, the most prominent ones being decreased weight gain and diminished muscle-skeletal development, which directly affect the carcass yield and reproductive aspects of replacement heifers [5,6,7].

Controlling this disease with anthelmintics is not, however, problem-free. The widespread use of anthelmintics, often without proper administration criteria, has led to the development of global anthelmintic resistance. Ivermectin (IVM)—widely used as an endectocide—is a clear example of this. The first reports of IVM resistance in cattle in Argentina occurred in 2001 [8,9], involving the genus *Cooperia*. Subsequent reports of anthelmintic resistance in cattle continued nationwide, with the latest survey reporting a 93.5% prevalence of IVM resistance in livestock farms across six provinces, once again involving *Cooperia* as the main genus [10].

Alongside the global evolution of anthelmintic resistance, various in vitro methods have been developed for its early detection, including the egg hatch test (EHT) [11], the larval migration inhibition test (LMIT) [12], the larval feeding inhibition test (LFIT) [13], and the larval development test (LDT) [14] and its variants [15]. These include the use of an agar matrix impregnated with the anthelmintic in a microtiter plate [16] or the replacement of *E. coli* with yeast extract, as introduced by Taylor (1990) [17] and later modified by Hubert and Kerboeuf (1992), who used Earle’s balanced salts, yeast extract, and bacteria as the nutritional medium [18].

Different parasite management strategies have also been investigated to delay the onset of, or even reverse, anthelmintic resistance, with parasite population replacement being one of the explored approaches [19,20,21,22]. The implementation of parasite population replacement has been reported mainly for small ruminants [19,20,21,22,23,24,25,26]. In cattle, to the authors’ knowledge, this strategy has only been investigated in Argentina through two scenarios. Briefly, the first involves creating a susceptible parasite refuge by introducing calves carrying non-resistant GIN during the summer, providing an infection source for calves weaned in autumn; the second method introduces untreated weaned animals carrying susceptible GIN to grazing areas with poorly resistant refuges, thereby increasing the susceptible parasite refuge. In both cases, this strategy has been reported as successful [27,28], representing a significant advance in the management of anthelmintic resistance.

This study aims to characterize field populations of *Cooperia* spp. resistant and susceptible to IVM using the micro-agar larval development test (MALDT) within the context of a parasite population replacement strategy in cattle.

## 2. Materials and Methods

### 2.1. Parasites

Three field isolates of *Cooperia* spp. were used in this study. These isolates originated from two different Cattle Production Sections of the Balcarce Experimental Station, National Institute of Agricultural Technology (EEA Balcarce-INTA), in the southeast of Buenos Aires Province, Argentina. The three isolates were as follows:

*Coop-S*: This IVM-susceptible isolate originated from the Cattle Production Section 6 (organic cattle section). It demonstrated 99.5% and 99.6% susceptibility to IVM based on a controlled efficacy test (CET) and a fecal egg count reduction test (FECRT), respectively.

*Coop-R*: This IVM-resistant isolate originated from the Cattle Production Section 7. It demonstrated 31% and 85% susceptibility to IVM as determined by CET and FECRT, respectively.

*Coop-PR*: This isolate originated from another area of the Cattle Production Section 7. It was obtained after the implementation of a parasite population replacement strategy, which consisted of introducing weaned calves naturally infected with the *Coop-S* isolate into the experimental pasture for one year. At the end of that period, the resulting *Cooperia* spp. population showed 87% and 91.4% susceptibility to IVM, according to CET and FECRT, respectively [28].

All isolates were individually maintained by artificial infections of 90–120-day-old Holstein-cross, parasite-naïve, calves. The animals were orally infected with 7000–9000 third-stage larvae (L3) of each isolate, which had been previously obtained by macro-coprocultures from the original field samples. The calves were housed individually, and fecal samples were routinely collected for the experiments. A detailed breakdown of the parasitic genera present in each inoculum is provided in Table 1.

### 2.2. Micro-Agar Larval Development Test (MALDT)

Due to the low solubility of IVM and the unavailability of IVM aglycone, a commercially available 0.5% eprinomectin (EPR) formulation (Eprinover^®^, Over, San Vicente, Argentina) was used. A stock solution was prepared by diluting 100 µL of the formulation in 9900 µL dimethyl sulfoxide (DMSO), followed by 12 serial dilutions in distilled water in order to obtain final drug concentrations ranging from 4.75 × 10^−8^ M–2.18 × 10^−11^ M (43.5 ng/mL–0.02 ng/mL).

The MALDT was conducted as per the method described previously [29]. In a 96-well microtiter plate, 2% bacteriological agar (Britania^®^, Lancashire, UK) impregnated with 12 µL of each EPR concentration was placed in each row of wells. As a control, agar with DMSO with a final concentration of 1% was used. Eggs from each isolate were recovered from calf feces as described previously [15]. The feces were filtered through 105 and 74 µm meshes, and the collected eggs were placed in Falcon tubes and centrifuged at 3000 rpm for 5 min. The supernatant was then removed, saturated saline solution was added, and the tubes were centrifuged again as before. The supernatant was then washed with water while filtering through a 37 µm mesh, thus retaining the eggs. Approximately 50–80 eggs in 10 µL of distilled water solution were added to each well and incubated in darkness for 24 h at 24–25 °C. After this period, 10 µL of culture medium containing yeast extract (Britania^®^), Earle’s salts (Sigma^®^, Livonia, MI, USA) [18], and amphotericin B (Calibiochem^®^, San Diego, CA, USA) were added to prevent contamination. The plates were covered and incubated for an additional 6 days at the same temperature. The test was stopped by adding one drop of iodine solution to each well. The eggs, L1/L2, and L3 of *Cooperia* spp. were then counted using an optical microscope. The MALDT was performed in quadruplicate across 15 time points for the *Coop-S* and *Coop-PR* isolates (n = 60 for each concentration) and 13 time points for *Coop-R* (n = 52 for each concentration).

### 2.3. Data Analysis

For each isolate, the percentage of fully developed L3 in each well was expressed as the relative percentage of L3 in the control well. Data were analyzed by a nonlinear regression model (dose–response curve normalized with variable slope) with 95% confidence intervals (95% CI) using Graph Pad Prism^®^ version 8.0.1 for Windows, GraphPad Software, Boston, MA, USA, www.graphpad.com (accessed on 1 October 2024). The EC50 value, EC90 value, EC99 value, and resistance ratio (RR) (EC-resistant isolate/EC-susceptible isolate) were calculated for each isolate. EC50, EC90, and EC99 are defined as the effective IVM concentration where development to the L3 stage is inhibited by 50%, 90%, and 99%, respectively. The coefficient of determination (R^2^ value) was also calculated to evaluate the goodness of fit of the study model.

## 3. Results

All isolates demonstrated an average of larval development above 93% (88–100%) in the control wells, except in those cases where eggs stored under anaerobic conditions for 1 to 3 days were used, in which case the development ranged from 64% to 88% regardless of the storage time. Thus, for subsequent tests, only freshly recovered eggs were used.

**Figure 1:**
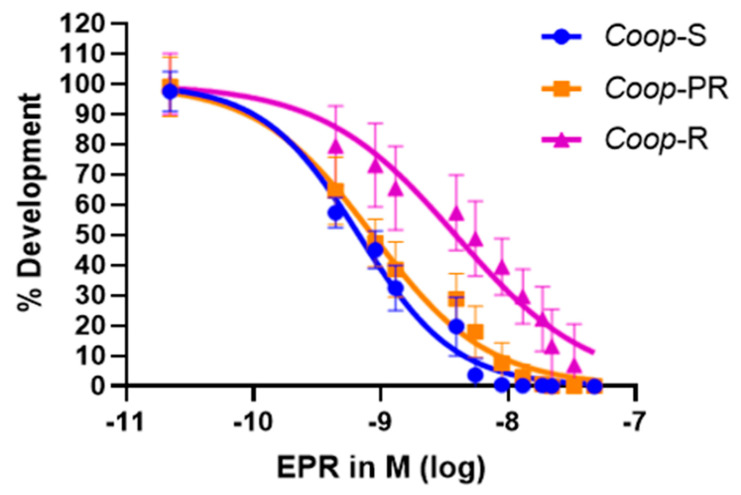
Dose–response for *Coop-S*, *Coop-R*, and *Coop-PR* isolates obtained from the MALDT (CI: 95%). EPR: Eprinomectin.

The dose–response curves obtained for each isolate (Figure 1) showed a shift to the right for the IVM-resistant isolate (i.e., a higher EC50), while the post-replacement isolate revealed a curve very similar to that of the susceptible one. The *Coop-S* isolate had an EC50 of 6.71 × 10^−10^ M (95% CI: 6.46 × 10^−10^ to 6.96 × 10^−10^), for the *Coop-R* isolate the EC50 was 3.88 × 10^−9^ M (95% CI: 3.55 × 10^−9^ to 4.20 × 10^−9^), and the *Coop-PR* isolate showed an EC50 of 8.62 × 10^−10^ M (95% CI: 8.21 × 10^−10^ to 9.04 × 10^−10^). Table 2 shows the EC90 and EC99 values of each isolate, their 95% confidence intervals and their respective R^2^.

The resistance ratio obtained for the EC50 between the *Coop-R* isolate and the *Coop-S* and *Coop-PR* ones was 5.78 and 4.5, respectively, while the resistance ratio between *Coop-PR* and *Coop-S* was 1.28. The same pattern of higher resistance ratios between *Coop-R* and *Coop-S* and *Coop-PR* and similar ratios between *Coop-PR* and *Coop-S* was observed for the EC90 and EC99 (Table 3).

## 4. Discussion

The MALDT has been reported as a useful tool for diagnosing anthelmintic resistance in ovine gastrointestinal nematodes by several authors [30,31,32,33], with a commercial kit, DrenchRite^®^ (Microbial Screening Technologies, Kemps Creek, Australia), available for diagnosing resistance in *Haemonchus contortus*. However, its use in cattle for the same purpose has only been reported once [34]. The EC50 values obtained in the present study were much lower than those reported by Demeler et al. [34]. This, perhaps, could be attributed to differences in testing conditions; while those authors used an LDT in a liquid medium and 48-well plates, in the present study, an MALDT on an agar base with 96-well plates was used. Different authors [29,32] have stated that the MALDT is more sensitive than other variants of the LDT, such as the one using a liquid medium, which could partly explain the lower EC50 values observed in the present study. However, it is interesting to note that the EC50-resistance ratio comparing IVM-resistant and -susceptible *Cooperia* spp. isolates was similar in both the present study and the previous one [34].

The resistance ratios showed that, in all cases, the resistant and susceptible isolates were clearly distinguishable. These findings are similar to those reported previously by this research group [35]. Moreover, the *Coop-PR* and *Coop-S* isolates showed minimal differences in susceptibility to IVM, which not only coincides with the results from the in vivo tests [36] but also corroborates the success of the population replacement strategy applied, given that the susceptibility of the parasite population increased from 31% to 87% in just one calf-rearing season on pasture.

The assay sensitivity has been reported to increase when resistance ratios were calculated based on the EC99, as it established more marked differences between isolates [31,37]. However, the opposite occurred in the present study; the resistance ratios based on EC90 and EC99 were not as effective in differentiating between susceptible and resistant isolates as the one based on the EC50. A similar observation was made by Dolinská et al. [32] when evaluating the potential of the LDT for detecting IVM resistance in *Haemonchus contortus* in sheep. Perhaps a small difference between EC50 and EC90 values, as recorded in the present study, could explain the lack of increased sensitivity when using EC90 or EC99. Since the only previous report with this type of in vitro test in *Cooperia* spp. in cattle only estimated the EC50 [34], the findings of the present study should be corroborated in further trials.

On the other hand, the MALDT has proven to be a robust and reliable test, as corroborated by the R^2^ values obtained, thus indicating that the model fits the data well, as previously found for this test in cattle [34].

Finally, it is noteworthy that this study used field isolates where *Cooperia* spp. was either the only genus present or the predominant one (>90%) in a mixed GIN infection. Despite this, the presence of other parasitic genera did not affect the test results, as indicated by the coefficients of determination obtained for the *Coop-R* and *Coop-PR* isolates. However, the influence of interactions between different GIN genera, as reported for sheep [16,17], should not be overlooked. This aspect will be important if the MALDT is to be adopted as a preferred method for early diagnosis of anthelmintic resistance in cattle, particularly because other genera, such as *Ostertagia*, are emerging as IVM-resistant as well (S. Fernández, personal communication).

## 5. Conclusions

The in vitro MALDT method has proven to be a useful tool for characterizing and differentiating the IVM-resistance/susceptibility status of *Cooperia* spp. isolates in cattle within the context of parasite population replacement. However, for it to become a preferred method of early anthelmintic resistance diagnosis in cattle, future work should consider mixed nematode populations commonly present in extensive systems and their interactions.

## Figures and Tables

**Table 1:** Parasitic genera composition (expressed as percentage values) of the inocula used for maintaining the three different isolates in artificially infected calves.

		Parasitic Genera (%)			
	Total L3	*Cooperia*	*Ostertagia*	*Haemonchus*	*Oesophagostomum*
***Coop-S***	9000	100	-	-	-
***Coop-R***	7970	90	7	2	1
***Coop-PR***	9850	90	8	-	2

Total L3: numbers of L3 present in the inoculum. *Coop-S*: susceptible isolate; *Coop-R*: resistant isolate; *Coop-PR*: isolate resulting from replacing the resistant parasite population by a susceptible one.

**Table 2:** Average values of EC50, EC90, and EC99 with their respective 95% confidence intervals (in brackets) as well as R^2^ values obtained in the MALDT for each *Cooperia* spp. isolate.

	*Cooperia* spp. Isolates		
	*Coop* *-S*	*Coop-R*	*Coop-PR*
**EC50**	6.71 × 10^−10^ M (6.46 × 10^−10^–6.96 × 10^−10^)	3.88 × 10^−9^ M (3.55 × 10^−9^–4.20 × 10^−9^)	8.62 × 10^−10^ M (8.21 × 10^−10^–9.04 × 10^−10^)
**EC90**	9.92 × 10^−11^ M (1.03 × 10^−10^–9.54 × 10^−11^)	2.7 × 10^−10^ M (2.94 × 10^−10^–2.47 × 10^−10^)	8.76 × 10^−11^ M (9.14 × 10^−11^–8.39 × 10^−11^)
**EC99**	1.23 × 10^−11^ M (1.38 × 10^−11^–1.09 × 10^−11^)	1.53 × 10^−11^ M (1.95 × 10^−11^–1.11 × 10^−11^)	7.29 × 10^−12^ M (8.32 × 10^−12^–6.27 × 10^−12^)
**R^2^**	0.96	0.82	0.93

*Coop-S:* susceptible isolate; *Coop-R*: resistant isolate; *Coop-PR*: isolate resulting from replacing the resistant parasite population by a susceptible one; EC50: average effective concentration; EC90 and EC99: concentrations required to affect 90% and 99%, respectively, of exposed individuals; R^2^: coefficient of determination.

**Table 3:** Resistance ratio (RR) obtained for each isolate in the MALDT using EC50, EC90, and E99 values.

RR	*Coop-R/Coop-S*	*Coop-PR/Coop-S*	*Coop-R/Coop-PR*
**EC50**	5.78	1.28	4.5
**EC90**	2.73	0.88	3.09
**EC99**	1.24	0.59	2.10

RR: relationship between EC (50, 90 or 99) values of IVM-resistant and IVM-susceptible isolates; EC50: average effective concentration; EC90 and EC99: concentrations required to affect 90% and 99%, respectively, of exposed individuals; *Coop-S*: susceptible isolate; *Coop-R*: resistant isolate; *Coop-PR*: isolate resulting from replacing the resistant parasite population by a susceptible one.

## Data Availability

The original contributions presented in the study are included in the article/Supplementary Materials; further inquiries can be directed to the corresponding authors.

## Supplementary Materials

The following supporting information can be downloaded at: https://www.mdpi.com/article/10.3390/pathogens13110952/s1, Table S1: Serial dilutions of eprinomectin 0.5% used for the MLDTA.
