# Supplementary material for: Use of the Micro-Agar Larval Development Test to Differentiate Resistant and Susceptible Cooperia spp. Isolates in Cattle Within the Context of Parasite Population Replacement
Source: Pathogens. 2024 Oct 31;13(11):952. doi: 10.3390/pathogens13110952 (PMC11597273; doi:10.3390/pathogens13110952)
Supplement: Supplementary file 1 [file pathogens-13-00952-s001.zip › pathogens-3260542-supplementary.pdf]

Table S1: Serial dilutions of eprinomectin (EPR) 0.5% used for the MLDTA

A stock solution was prepared by diluting first 100 µl of the EPR formulation in 9900 µl of DMSO, then 20 µl of the EPR+DMSO solution was diluted in 9980 µl of distilled water. From this stock solution, serial dilutions in distilled water were prepared so the following concentations of EPR were used en each of the MLDTA:

Dilution 1: 43.5 ng/ml (4.75 x 10^{-8} M)
Dilution 2: 30 ng/ml (3.3 x 10^{-8} M)
Dilution 3: 20 ng/ml (2.2 x 10^{-8} M)
Dilution 4: 17 ng/ml (1.85 x 10^{-8} M)
Dilution 5: 12 ng/ml (1.3 x 10^{-8} M)
Dilution 6: 8.13 ng/ml (8.9 x 10^{-9} M)
Dilution 7: 5.01 ng/ml (5.5 x 10^{-9} M)
Dilution 8: 3.49 ng/ml (3.9 x 10^{-9} M)
Dilution 9: 1.18 ng/ml (1.3 x 10^{-9} M)
Dilution 10: 0.82 ng/ml (9 x 10^{-10} M)
Dilution 11: 0.4 ng/ml (4.4 x 10^{-10} M)
Dilution 12: 0.020 ng/ml (2.2 x 10^{-11} M)
